# Blocking leukotrienes optimize asthma control: The BLOC survey

**DOI:** 10.4103/1817-1737.33696

**Published:** 2007

**Authors:** Majdy M. Idrees, Mohamed S. Al Moamary

**Affiliations:** Riyadh Military Hospital, Riyadh, Saudi Arabia; * *King Saud bin Abdulaziz University for Health Sciences and King Abdulaziz Medical City, Riyadh, Saudi Arabia*

**Keywords:** Asthma, inflammation

## Abstract

**Objective:**

The aim of this study was to evaluate asthma control after the introduction of a leukotriene modifier (Montelukast), in addition to the current controller asthma therapies, in patients with inadequately controlled mild-to-moderate persistent asthma. Asthma control and patient perception were assessed prior to, and 4 weeks after, the introduction of Montelukast, and the pre-introduction and post-introduction results were compared.

**Materials and Methods:**

A cross-sectional, observational study collected information on 1,490 eligible adult asthmatic patients in Saudi Arabia. The eligibility criteria included patients aged 15 years or more with symptomatic mild-to-moderate persistent asthma despite treatment with inhaled corticosteroids with or without long-acting beta agonist; also, the patient should attend the initial visit and follow-up visits after at least 4 weeks.

**Results:**

Of the 1,490 eligible patients, 79.5% received inhaled corticosteroids alone, and the remaining 20.5% received combination of inhaled corticosteroids and long-acting bronchodilator. Despite the treatment with daily controller medications, asthma symptoms persisted in more than two-thirds of the study population. Upon adding Montelukast, more than 80% of patients reported improvement in symptoms, which was consistent in all patients irrespective of corticosteroid type or dose (stratum) or the addition of long-acting β2-agonist. At the follow-up visit, 92.2% of patients reported that they felt better on Montelukast.

**Conclusion:**

Leukotriene modifier Montelukast has significant additive benefits in the management of patients who suffer from mild-to-moderate asthma and who are inadequately controlled on inhaled corticosteroids therapy with or without long-acting bronchodilator.

Asthma is a complex respiratory disorder characterized by reversible airflow obstruction and bronchial hyper-reactivity.[1] In recent years, the understanding of the mechanism underlying allergic asthma has grown, and this has resulted in complex paradigms to define the immunobiology of the disorder.[1] These paradigms conceive of asthma as a disorder with complex genetic and environmental interactions that affect the developing immune system and ultimately result in the episodic release of procontractile mediators, including leukotrienes and prostaglandins, causing susceptible individuals to wheeze.[2]

Not all asthmatics can be adequately controlled with high-dose inhaled corticosteroid (ICS) alone or in combination with long-acting bronchodilators (LABA). The Global Initiatives for Asthma (GINA) recommended leukotriene modifiers as add-on therapy in patients with uncontrolled moderate persistent asthma and also as alternative to long-acting bronchodilator in moderate persistent asthma.[1] This was further stratified by the newly released update from GINA in November 2006, where more evidence is available with respect to efficacy of leukotriene modifiers in patients with uncontrolled asthma.[2–4]

The objective of this observational cross-sectional survey was to evaluate asthma control and patient perception after the introduction of a leukotriene modifier, Montelukast, in addition to the current controller asthma therapies, in patients with inadequately controlled mild-to-moderate persistent asthma. Asthma control and patient perception were assessed prior to, and 4 weeks after, the introduction of Montelukast, and the pre-introduction and post-introduction results were compared.

## Materials and Methods

This observational, cross-sectional, prospective survey recruited asthmatic patients from the general practice setting in the major cities of the Kingdom of Saudi Arabia: Riyadh, Jeddah, Mecca, Medina, Dammam, Abha, Kamis Mushate, Jubail, Qasim, Yanbu and Jizan. The study was conducted in the period between December 2003 and July 2004. Recruited patients were above the age of 15, with mild-to-moderate persistent asthma, who were still uncontrolled despite ICS therapy at any dose with or without LABA. Patients were selected by physicians in the general practice setting, where both parties volunteered and were unpaid. Patients on Montelukast were excluded. It was mandatory for the patients to attend the initial first and second visits and complete the questionnaires in order to be eligible for the final analysis.

The patients were evaluated twice. At the first visit, the current asthma symptoms were recorded and described by (a) asthma-related difficulty in sleeping, (b) early morning awakening, (c) limitation of activities and (d) short-acting β2-agonist (SABA) use twice or more per week. Patients were informed about the cross-sectional survey objectives and the leukotriene modifiers as one of the recommended standard option for a patient with uncontrolled mild and moderate persistent asthma.[1] All patients were started on oral Montelukast 10 mg daily at the initial visit.

The second visit was scheduled at the discretion of the participants. However, it was recommended to be performed at least 4 weeks after the first visit. At the second visit, the patients were asked to fill in a report, commenting on the change of their symptoms (Less, Unchanged, More) in regard to (a) early morning awakening, (b) difficulty with sleep due to asthma, (c) limitations of activities due to asthma and (d) SABA use twice or more times a week. Patients were classified into three strata based on the dose of ICS: stratum I with ICS ≤400 μg/day, stratum II with dose between 401 and 800 μg/day and stratum III with dose ≥800 μg/day.

‘*P*’ value was calculated by Chi-square test. *P* value more than 0.05 was considered not significant. *P* value of less than 0.05 but more than 0.01 was rated as significant, 0.01 to 0.001 as highly significant and <0.001 as very highly significant.

## Results

Of the 1,687 patients recruited in the study, 1,490 patients (88.3%) were eligible and attended the second visit. Table 1 shows the demographic data of patients at the initial visit, their prescribed treatment and stratum based on ICS dose. Table 2 shows the symptoms reported by the patients at the initial visit with reference to the type of controller therapy. Patients on combined ICS and LABA reported less symptoms compared to ICS alone except for limitation of activities, where no significant difference was observed. The symptoms reported with reference to ICS type and strata are reported in Table 3.

**Table 1:** The demographic data of patients at the initial visit and their prescribed treatment

Type and mean daily dose of ICS	No.	Percentage	Mean daily ICS dose (μg/day)
Age	37.8 (± 8.6)		
Male	615	41.3	
Female	875	58.7	
Patients on ICS alone	1184	79.5	
Patients on combination of ICS and LABA	306	20.5	
Patients on			
Beclomethasone	264	17.7	537 (± 121).
Patients on Budesonide	620	41.6	590 (± 107).
Patients on Fluticasone	606	40.7	542 (± 93).
Patients per steroids stratum:			
No. of patients on stratum I (≤ 400 μg/day)	568	38.1	
No. of Patients on stratum II (401 - 800μg/day)	509	34.2	
No. of patients on Stratum III (≥800 μg/day)	413	27.7

ICS - Inhaled corticosteroid

**Table 2:** Symptoms reported at the initial visit with reference to type of controller therapy

	All patients		ICS alone		ICS + LABA		*P* value
				
	No.	%	No.	%	No.	%	
Number of patients	1490		1184			306		
Early morning awakening	1084	72.8	896	75.7	188	61.4	< 0.05
Difficulty in sleeping	1127	75.6	925	78.1	202	66.0	< 0.001
Limitation of activities	1207	81.0	965	81.5	242	79.1	NS
SABA use ≥ twice a week	1131	75.9	922	77.0	209	68.3	< 0.001

LABA - Long-acting bronchodilators, ICS - Inhaled corticosteroid

**Table 3:** Symptoms reported at the initial visit with reference to inhaled corticosteroid type and stratum

	Inhaled corticosteroid type						Stratum type					
			
	Beclomethasone		Budesonide		Fluticasone		Stratum 1		Stratum 2		Stratum 3	
							
	No.	%	No.	%	No.	%	No.	%	No.	%	No.	%
Number of patients	264	100	620	100	606	100	568	100	509	100	413	100
Early morning awakening	202	76.4	466	75.2	440	72.6	426	75.0	376	73.9	282	68.3
Difficulty in sleeping	196	74.2	481	77.6	450	74.3	439	77.3	381	75.0	306	74.1
Limitation of activities	228	86.4	499	80.5	480	79.2	465	81.9	401	78.8	341	82.6
SABA use ≥ twice a week	212	80.3	472	76.2	436	71.9	440	77.5	356	70.1	324	78.5

**Figure 1:**
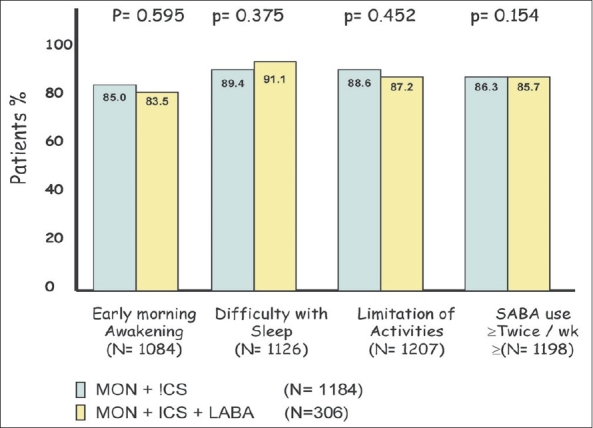
Observed improvement in symptoms at the second visit after adding Montelukast

At the second visit after adding Montelukast, 1,296 out of 1,490 (87%) patients reported improvement. The observed improvement was consistent for all symptoms addressed, i.e., early morning awakening, difficulty in sleeping, limitations of activities and SABA use twice or more times a week [Figure 1]. Figure 1 also shows that the observed benefits by adding Montelukast were not significantly different, whether the patients were on ICS alone or on combination of ICS and LABA.

**Figure 2:**
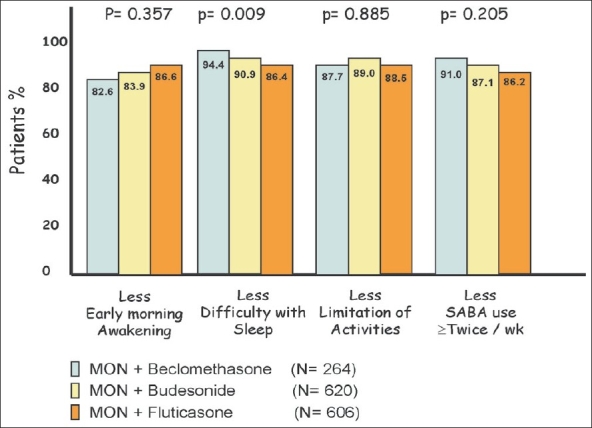
Improvement in symptoms after adding Montelukast, based on ICS type

**Figure 3:**
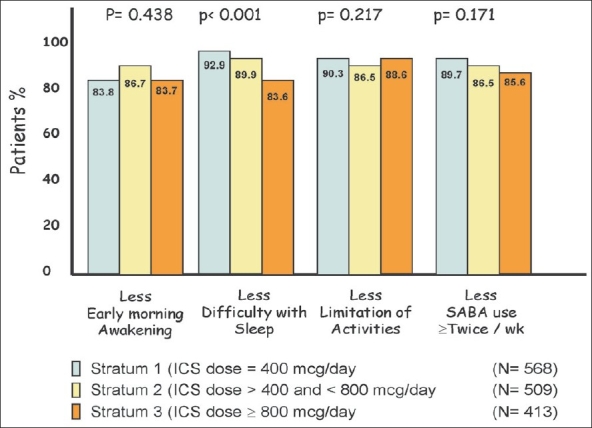
Improvement in symptoms after adding Montelukast, based on ICS dose

At the second visit after adding Montelukast, there was reported symptomatic improvement regardless of the type of ICS. The improvement was universal to all the reported symptoms by the patients [Figure 2]. However, there was significant difference between Fluticasone and the other ICS when combined with Montelukast with respect to improvement in the symptom ‘difficulty with sleep’ (*P* = 0.009). Figure 3 shows that the reported symptomatic improvement was consistent in all strata and ICS doses. There was less improvement in the symptom ‘difficulty in sleeping’ in patients of stratum 3 when Montelukast was combined with high dose of ICS (*P* < 0.001).

When Montelukast was combined with ICS alone, 1,103 patients (93.2%) reported feeling better at the second visit, while 271 patients (88.4%) felt better after adding Montelukast to the combination of ICS and LABA (*P* = 0.007). Table 4 shows that overall patient perception of improvement was consistent with reference to different types of ICS and strata.

**Table 4:** Overall patient perception of improvement with reference to inhaled corticosteroid type and stratum

	Better		Same		No change	
			
	No.	%	No.	%	No.	%
ICS type:	264		620		606	
Beclomethasone	243	92.2	14	5.1	7	2.7
Budesonide	582	93.9	24	3.8	14	2.3
Fluticasone	554	91.4	42	6.9	10	1.7
Strata based on						
Inhaled corticosteroid dose:						
Stratum 1 (≤ 400 mcg)	538	94.9	28	4.9	2	0.2
Stratum 2 (400 - 800 mcg)	464	91.3	39	7.5	6	1.2
Stratum 3 (≥ 800 mcg)	370	89.7	31	7.3	12	3.0

## Discussion

Leukotrienes are considered to be important components in the treatment of asthma inflammation as some asthmatics cannot be controlled by other modalities like ICS and LABA.[2,5] The cysteinyl leukotrienes (CysLT), LTC4, LTD4 and LTE4 have been shown to be essential mediators in asthma, making them obvious targets for therapy. These cysteinyl leukotrienes, previously known as the slow-reacting substance of anaphylaxis (SRS-A), mediate many of the features of asthma, including bronchial constriction, bronchial hyper-reactivity, edema and eosinophilia.[2,5] Data show that selective cysteinyl leukotriene receptor antagonists (CysLTRAs) effectively reverse these pathologic changes. However, corticosteroids do not block the leukotriene-mediated pathway of inflammation.[2,5–7] Corticosteroids do not inhibit the production of CysLTs *in vivo,* suggesting that CysLTRAs and corticosteroids affect different targets. The anti-inflammatory properties of CysLTRAs seem to be additive to those of β2-agonists and corticosteroids.[8,9]

In our survey of 1,490 patients with persistent asthma symptoms despite regular use of ICS with or without LABA, Montelukast was added to the standard treatment as per GINA guidelines.[1] Of the patients having symptoms at the start of the study, adding Montelukast led to beneficial results, and the vast majority of patients reported improvement in sleep, less frequent early morning awakening, better ability to perform daily activities and decreased need for rescue medication, as well as improved quality of life.

The anti-inflammatory properties of Montelukast seem to be additive to those of ICS. The complementary benefits of Montelukast are due to blockade of the leukotriene pathway - key mediators in asthmatic inflammation that are not blocked by steroids.[5] Recent data has clearly shown that airways inflammation in asthma improved but persisted despite treatment with ICS or oral prednisolone.[10] Furthermore, treatment with ICS has no significant effect on the leukotrienes' inflammatory mediators in asthma.[2,11,12] It has been found that at least dual pathways of inflammation exist in asthma - the prostaglandin cytokines pathway and leukotrienes pathway.[13] Different clinical trials have shown the benefit of adding anti-leukotrienes to ICS, confirming the presence of dual pathways of inflammation.[14–16] All these data suggest that Montelukast is an important therapeutic agent in the management of uncontrolled asthma as an add-on therapy to ICS.

The results in our study were consistent with those of Malonne *et al.* in the nationwide Belgium ASTHMA survey among general practitioners (GPs) to evaluate the impact of Montelukast on the control of asthma symptoms, after at least 4 weeks of treatment.[2] Of the patients having symptoms at the start of the study, 87% reported amelioration in sleep while on Montelukast therapy; 80%, less frequent early morning awakening; 85%, better ability to perform daily activities; and 77%, decreased need for rescue medication.[2] Moreover, a recent multicenter study evaluated the effects of addition of Montelukast on patients with mild-to-moderate asthma with seasonal rhinitis who were maintained on ICS (alone or in combination with LABA). Upon completing 12 months of consecutive Montelukast, there was significant reduction in asthma attacks, asthma-related emergency visits, hospitalization and oral steroids use.[17]

Our study is an observational survey, and its results should be considered as such. The important limitation for all observational studies is the lack of control and blind randomization. In our study, we did not account for patient's adherence to therapy, the presence of comorbid conditions such as allergic rhinitis, gastroesophageal reflux or the presence of any occupational or environmental triggers. However, despite these limitations, the importance of observational data is the reflection of reality, far from the ‘unreal’ strict patients' monitoring that usually takes place in randomized controlled trials.
